# Development and Validation of a DNA Methylation-related Classifier of Circulating Tumour Cells to Predict Prognosis and to provide a therapeutic strategy in Lung Adenocarcinoma

**DOI:** 10.7150/ijbs.75284

**Published:** 2022-08-01

**Authors:** Xuyu Gu, Xianting Huang, Xiuxiu Zhang, Cailian Wang

**Affiliations:** 1 School of Medicine, Southeast University, Nanjing 210009, China.; 2 Nanjing Medical University, Nanjing, 210011, Jiangsu, China; Department of Oncology, Jiangyin People's Hospital, Jiangyin, 214400, China.; 3 Department of oncology, Zhongda Hospital, School of Medicine, Southeast University, Nanjing 210009, China.

**Keywords:** Lung adenocarcinoma, Circulating tumour cell, DNA methylation, Risk model, Clinical outcomes

## Abstract

**Background:** A significant factor influencing the prognosis of lung adenocarcinoma (LUAD) is tumor metastasis. Studies have shown that abnormal DNA methylation in circulating tumor cells (CTCs) is associated with tumour metastasis. Based on the genes expressed in CTCs that play an important role in DNA methylation, we hope to build a risk model to predict prognosis and provide a therapeutic strategy in LUAD.

**Methods:** The CTC sequencing data for LUAD were obtained from GSE74639, which contains 10 CTC samples and 6 primary tumour samples. To carefully assess the clinical value, functional status, involvement of the tumor microenvironment (TME) based on the risk model, and genetic variants based on based on data from The Cancer Genome Atlas (TCGA) and the Gene Expression Omnibus (GEO), a reliable risk model was successfully built.

**Results:** Three differentially methylated genes (DMGs) of CTCs for LUAD, including mitochondrial ribosomal protein L51 (MRPL51), STE20-like kinase (SLK), and protein regulator of cytokinesis 1(PRC1), were effectively used to construct a risk model. Both the training and validation cohorts' stability and accuracy of the risk model were evaluated. Each patient in the TCGA-LUAD cohort received a risk score, and based on the median score, they were divided into high- and low-risk groups. The tumors in the high-risk group in this study were classified as "cold" and immunosuppressed, which may be linked to a poor prognosis. The tumors in the low-risk group, however, were deemed "hot" and had immune hyperfunction linked to a positive prognosis. Additionally, patients in the low-risk group showed greater sensitivity to immunotherapy than those in the high-risk group.

**Conclusions:** Based on DMGs of CTCs from LUAD, we successfully developed a predictive risk model and discovered differences in biological function, TME, genetic variation, and clinical outcomes between those at high and low risk group.

## Introduction

The most prevalent and fatal kind of non-small cell lung cancer (NSCLC) is lung adenocarcinoma (LUAD) 1. Each year, there is an increase in the incidence and mortality of LUAD. It has a 15% 5-year survival rate, on average 2. The prognosis of LUAD is still a significant clinical problem and remarkably varies between different LUAD patients 3. In recent years, an increasing number of prognostic biomarkers for LUAD have been found by analyzing clinical information combined with expression profiles in public databases. For patients with advanced cancer, prognostic risk models generated from public databases showed tremendous potential in these research 4. However, whether these prognostic models can be successfully applied to clinical practice has not been confirmed. Thus, it is necessary to continue to mine genes and polygenic signatures associated with the prognosis of patients with LUAD.

Circulating tumor cells (CTCs) are cells that leave malignant tumors, travel through the blood, and become vital components of the tumor metastasis process 5. According to research, CTCs change DNA methylation to facilitate metastatic seeding 6. The epigenetic alteration of DNA methylation in human malignancies has received the greatest attention. It was discovered that the DNA hypermethylation of tumor suppressor genes (SOX17, CST6, and BRMS1) was a characteristic of CTCs in patients with breast cancer 7. According to research, the SOX17 tumor suppressor gene was shown to be hypermethylated in CTCs from breast cancer patients 8. Although it is not obvious if epigenetic markers in CTCs are therapeutically meaningful as biomarkers, cancer proliferation and metastasis may be connected to aberrant alterations in the epigenetic machinery, according to these studies 9.

In this study, we set out to build a risk model using multivariate Cox regression and random forest to predict the patient prognosis and to inform clinical decision-making in order to explore how DNA methylation in CTCs may be used to forecast a patient's prognosis, immunological status, and clinical response to treatment in patients with LUAD.

## Materials and Methods

### Data acquisition

Data from primary tumour samples (n = 6) and CTC samples (n = 10) of lung cancer are included in the dataset GSE74639, which was retrieved from the GEO database at http://www.ncbi.nlm.nih.gov/geo/. The TCGA GDC portal (https://www.tcga.org) was used to download the RNA-Seq data for 492 LUAD patients with full clinical data, and fragments per kilobase per million (FPKM) were then converted to transcripts per million (TPM) for data training. The methylation, Muctec2 somatic mutation, and copy number variation (CNV) data of 492 pertinent patients were acquired for the Illumina Human Methylation 450 BeadChip of LUAD (https://xena.ucsc.edu/). Validation data were retrieved from the GEO database, including GSE72094 (n=398) from the Rosetta/Merck Human RSTA Custom Affymetrix 2.0 platform and GSE42127 (n=132) from the Illumina HumanWG-6 v3.0 expression BeadChip platform. The three main cohorts had 1022 samples altogether. Following the exclusion of patients who did not meet the requirements, individuals with full clinical information (Stage, Follow-up Information, Age, and Gender) were selected for this research. The precise clinical characteristics are detailed in Table 1. To predict the immunotherapy response, GSE126044 and GSE135222 microarrays with treatment information from NSCLC patients receiving anti-PD-1/PD-L1 therapy were gathered. The IMvigor210 dataset contains 298 urothelial carcinoma patients who were treated with anti-PD-L1. It was created using open-source, well-documented software from http://research-pub.gene.com/IMvigor210CoreBiologies. Somatic mutation data for the TCGA-LUAD cohort were obtained in MAF format from UCSC Xena (https://xena.ucsc.edu/). Using the R package “maftools”, we explored the types and rates of DNA mutations. GISTIC 2.0 was used to gather and preprocess copy number variation (CNV) data from the TCGA-LUAD cohort. CNV amplification was defined by score >0.2, while depletion was defined by score < -0.2.

### Screening of methylation-driven genes

Differentially expressed genes (DEGs) between CTC samples and main tumor samples were retrieved using the R package “limma” at a significance threshold of p<0.05. Additionally, the TCGA-LUAD cohort's metastatic and non-metastatic samples (verified with follow-up data) were compared to obtain DEGs with p<0.05. Candidate DEGs were created by intersecting the two sets of DEGs. Additionally, by examining the correlation between gene expression and methylation level using R<0 and p<0.05 as criteria, differential methylation-driven genes (DMGs) were found.

### Construction of risk model

The TCGA-LUAD cohort (n=492) served as the training set. DMGs were examined using univariate Cox regression and the log-rank test to identify predictive genes. To extract genes with a significance score >0, the random survival forests-variable hunting (RSFVH) method was computed. The Gaussian mixture model-based hierarchical clustering method was used to obtain and categorize a number of gene combinations (GMM). To assess the prognostic potency of the gene combinations, the area under the receiver operating characteristic curve (ROC-AUC) was calculated. The simplest model with the highest AUC score was found to be the best prognostic model. As a result, the following was the construction of a multivariate Cox regression model:



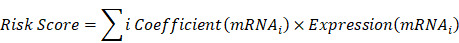



A higher C-index indicates better performance. The concordance index (C-index), which measures how well the model performed in both the training and test sets, was computed using the R package “survcomp”. Based on the median risk score obtained from all patient samples, risk groups with high or low risk were given. The prognostic utility of the model was assessed using Kaplan-Meier (KM) curves, univariate and multivariate Cox regression, and time-dependent ROC (tROC).

### Functional enrichment and immune infiltration analyses

In order to evaluate the activities of biological pathways in the samples, including cytolytic activity, myeloid inflammation, and other immune-related pathways, we performed a single-sample gene set enrichment analysis (ssGSEA) using the R package “gsva” based on previously reported molecular markers 10-13. Furthermore, GSEA was performed comparing the high-FRS and low-FRS groups, and significant KEGG pathways were identified using the standard p<0.05 criterion. Additionally, the Metascape (http://www.metascape.org) database was used to obtain functional enrichment of genes. To assess the amount of infiltration of 22 distinct immune cell types, the abundances of immune cell infiltrate in tumor samples were estimated using the R package “CIBERSORT” 14. The estimate algorithm was used to assess the immunological activity and tumor purity of the samples 15.

### Comparison of genomic variation landscapes between the two groups

The R package “maftools” was used to manipulate the mutation data in order to examine the differences in mutation loads between the two groups. After determining the total number of mutations in the samples, genes with a minimum number of mutations greater than 30 were identified. After the chi-square test was done to analyze the differences in mutation frequencies between the high- and low-risk groups, the results were presented using maftools 16. CNV data were processed using the Gistic 2.0 webtool in Genepattern. Subsequently, significantly amplified and missing chromosomal segments were identified, and differences in CNVs on the chromosomal arms were assessed. Finally, using the R package “ggplot2”, these CNV results were visualized.

### Prediction of potential small-molecule drugs and chemotherapy resistance

Potential effective small-molecule drugs were retrieved from the CTRP2.0 and PRISM databases. A ridge regression model was developed using TCGA transcriptome data to assess drug sensitivity in high- and low-risk groups. The Genomics of Drug Sensitivity in Cancer (GDSC) and the R package “pRRophetic” were used to select five first-line medications for LUAD, including cisplatin, docetaxel, gemcitabine, paclitaxel, and vinorelbine. The half maximum inhibitory concentration (IC50) of each of the five drugs for each sample was calculated using the ridge regression approach in order to assess the chemotherapeutic resistance of the high- and low-risk groups. In order to evaluate the accuracy, ten-fold cross-validation was used. In CMap (https://clue.io/), which found potential compounds linked to these DEGs, the DEGs between the high-risk and low-risk groups were employed as the targets of prospective small-molecule therapies. By using gene expression patterns to anticipate medications, this approach can also help identify the modes of action (MoA) of substances that are a part of important biochemical processes. Compounds with an enrichment score < -95 were considered potential therapeutic agents.

### Prediction of immunotherapy response

TIDE scores were computed for patients in each group to determine how well they responded to anti-PD-1 and anti-CTLA4 therapy 17,18. Unsupervised subclass mapping (https://cloud.genepattern.org/gp/) was adopted to analyse the similarity between the high- and low-risk patient data and the data from a published dataset consisting of 47 patients responding to anti-PD-1 and anti-CTLA4 therapy 19,20. This was done in order to evaluate the effectiveness of the immunotherapy. IMvigor210, GSE126044, and GSE135222 datasets were also used to test the risk score for immunotherapy response's prediction abilities.

### Bioinformatics and statistical analysis

R 4.0.4 was performed, enabling the creation of graphs and statistical analysis. For comparisons involving only two groups, the Wilcoxon test was used, while the Kruskal-Wallis test was applied for comparisons involving three or more groups. The chi-square test was used to compare proportional differences. The survival curves were produced using a Kaplan-Meier plotter. To investigate the relationships between gene expression, tumor mutation burden (TMB), and microsatellite instability (MSI), Spearman correlation analysis was used. Using Pearson's test, further correlation studies were carried out. The significance of the statistical difference was assessed using the log-rank test. The tROC curve was generated using the R package “survivalROC”, and the predictive power was assessed using the AUC. Using the R package “survival”, univariate and multivariate Cox regression analyses were conducted. The nomogram was produced using the R package “rms”. P<0.05 in the two-tailed test was regarded as statistically significant without the need for a formal declaration.

## Results

### Identification of differential methylation-driven genes

**Figure 1:**
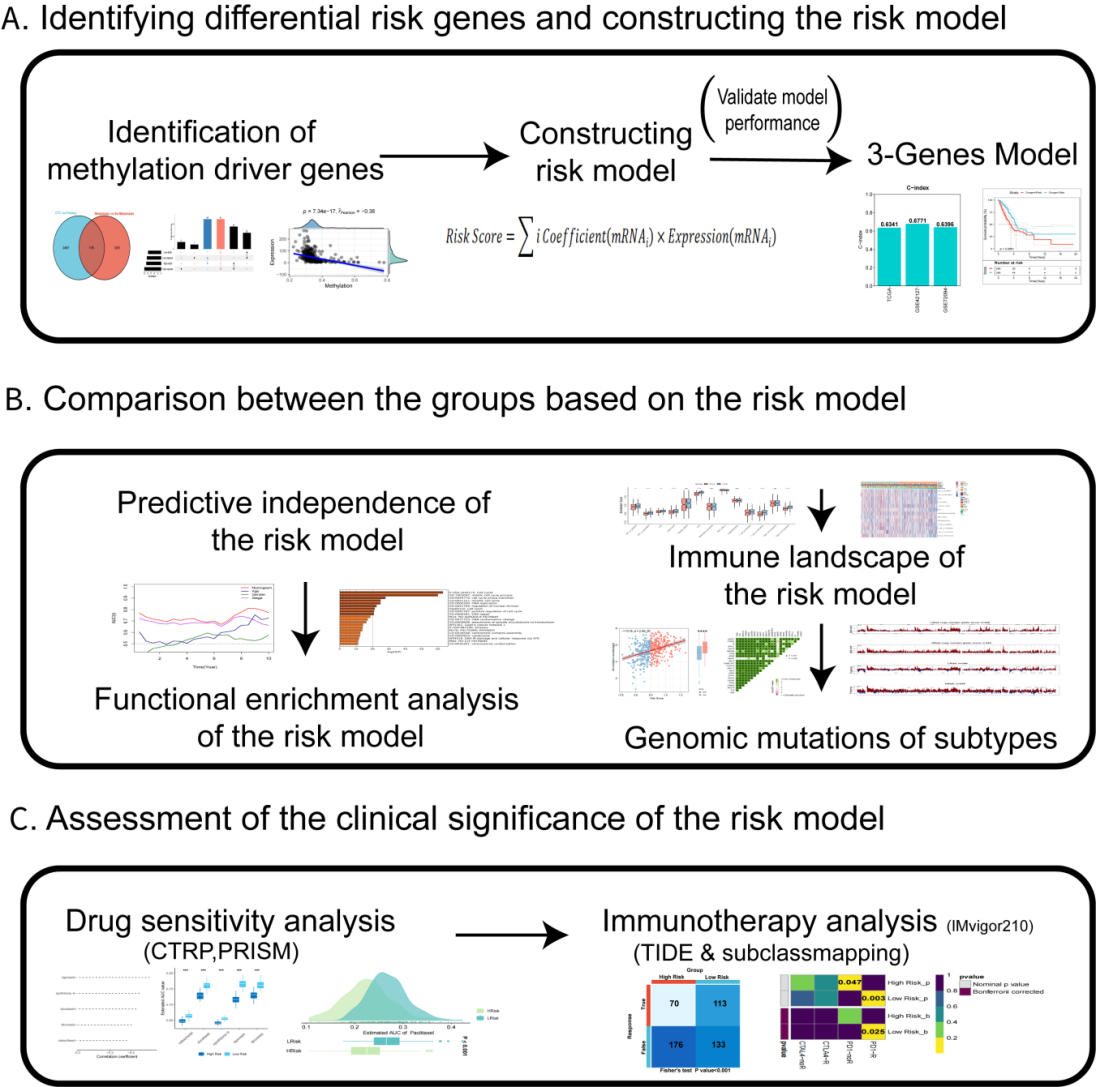
** The design and process of our study are shown in the flow chart.** In the present study, We first identified differentially expressed genes in CTC and *in situ* tumors based on dataset GSE74639 and TCGA-LUAD dataset in metastatic patients versus non-metastatic patients, and obtained methylation probe information of differential genes to identify differentially methylated driver genes. Then, A risk model was constructed which comprised of three DMGs in CTCs of LUAD. Next, we systematically assessed the prognostic model's stability and accuracy in both the external validation and the training cohorts. The biological functions, TME, and genomic variations in the prognostic model were evaluated in detail. At last, the value of the prognostic model was determined and its clinical applicability in chemotherapy and immunotherapy of LUAD was evaluated.

**Figure 2:**
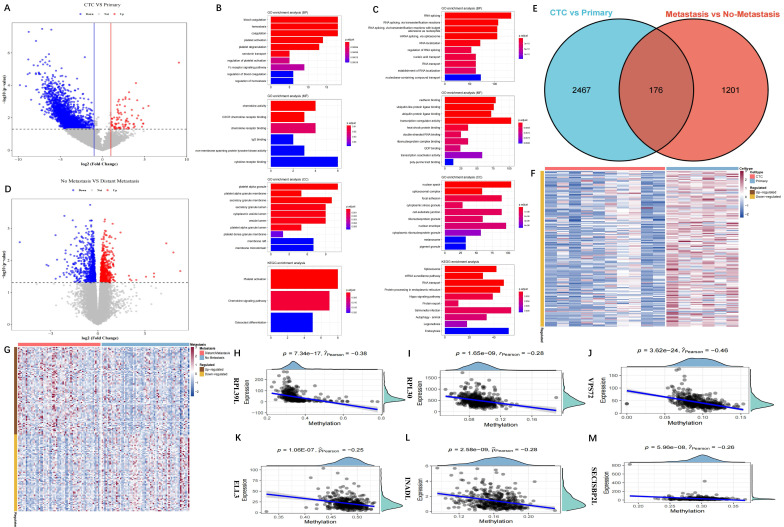
** Identification of methylation-driven DEGs. (A)** Volcano map of the DEGs between CTC and primary samples from GSE74639 dataset. There were 2,643 DEGs, including 92 genes up-regulated in CTC samples; **(B)** Functional enrichment analysis for the 92 genes up-regulated in CTC samples; **(C)** Functional enrichment analysis for the genes up-regulated in primary samples (BP, MF, CC, KEGG); **(D)** Volcano map of the DEGs between metastatic and non-metastatic samples from the TCGA-LUAD dataset. There were 1,377 DEGs, including 772 genes up-regulated in metastatic samples and 605 genes up-regulated in non-metastatic samples; **(E)** Venn diagram of the 2,643 DEGs in CTC vs primary and 1,377 DEGs in metastatic vs non-metastatic. There were 176 overlapping DEGs; **(F)** Transcriptome profile of the DEGs from GSE74639; **(G)** Transcriptome profile of the DEGs from TCGA-LUAD; **(H-M)** Six representative methylation-driven genes, including RPL39L (H), RPL30 (I), VPS72 (J), ELL3 (K), INADL (L) and SECISBP2L (M).

Figure 1 displays our study's design. A total of 2,643 DEGs were extracted between CTCs and primary tumour samples from the GSE74639 dataset. Among them, 92 DEGs significantly upregulated in CTC samples (Figure 2A) were found to be enriched in the platelet and coagulation pathways (Figure 2B). Our study found 2,551 DEGs that were significantly upregulated in primary tumour samples. They were mainly involved in RNA splicing and nucleic acid transport (Figure 2C). To obtain DEGs related to metastasis, metastatic and non-metastatic tissues in the TCGA-LUAD cohort were downloaded and analysed; 1,377 DEGs were identified, and 772 and 605 DEGs were up- and downregulated upon metastasis, respectively (Figure 2D). The 2,643 DEGs and 1,377 DEGs were intersected to obtain 176 overlapping DEGs associated with metastasis (Figure 2E). Methylated probe data were obtained from 158 genes out of the 176 genes. Transcriptome data of the 158 genes in the GSE74639 (Figure 2F) and TCGA-LUAD cohorts (Figure 2G) were visualized by heatmaps. 36 potential DMGs were found using correlation analysis with R0 and p0.05 (Table S1), and the 6 representatives are displayed using scatter plots (Figure 2H-M).

### Construction of the risk score model

**Figure 3:**
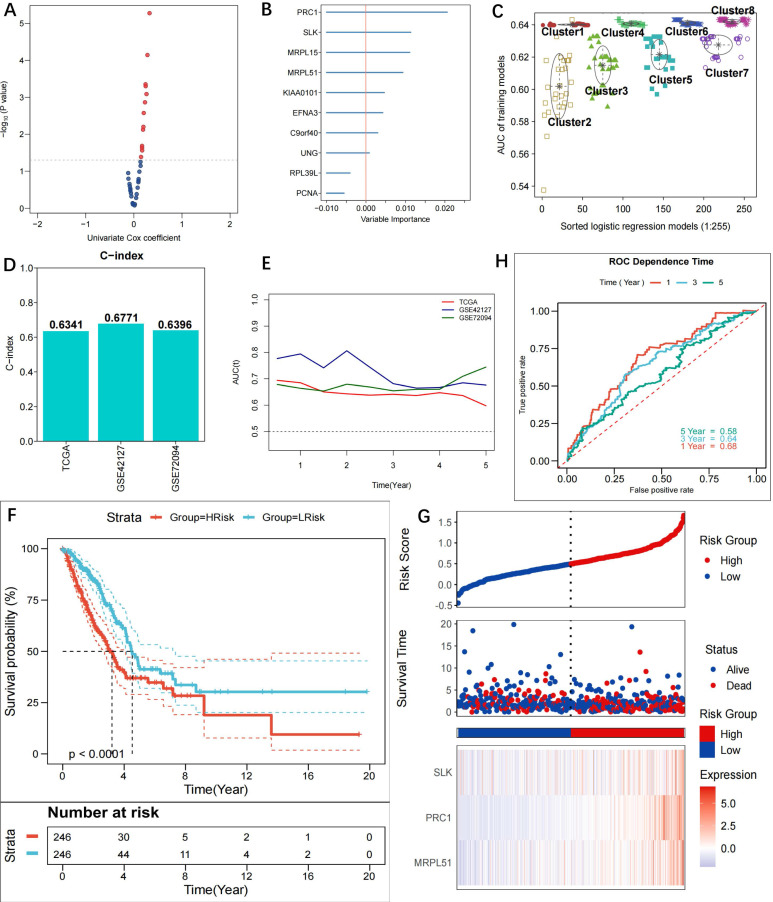
** Construction of prognostic riskscore model based on methylation-driven genes.** Scatter plot showing the 10 candidate prognostic genes obtained using uni-variate COX regression analysis (p<0.05); **(B)** Forest plot showing the importance of the 10 candidate genes, with the RPL39L and PCNA (importance score <0) genes eventually excluded to increase model stability; **(C)** Different GMMs based on clustering analysis and gene combinations in Cluster 8 identified as optimal in prognosis; **(D)** C-indexes of the model in TCGA-LUAD (0.6341), GSE42127 (0.6771) and GSE72094 (0.6396); **(E)** tROC curves for 1-, 3- and 5-year survival in TCGA and GEO cohorts. The 3-gene model (MRPL51, SLK, PRC1) was detected to be prognostic for OS of LUAD patients; **(F)** KM curves for the model in TCGA-LUAD cohort. Patients in the high-risk group experienced a significantly shorter survival time than patients in the low-risk group (p<0.0001); (G) Riskplot of TCGA patients in the high-risk group and expression of three model genes in high- and low-risk groups; **(H)** ROC curves for 1-, 3- and 5-year survival in the TCGA cohort (AUC: 0.68, 0.64, 0.58).

The 36 methylation-driven DMGs were subjected to univariate Cox regression analysis and the log-rank test (Figure 3A, Table S2), and 10 DMGs with prognostic value were discovered (p<0.05). The importance of the 10 genes was estimated using the random forest algorithm. RPL39L and PCNA were shown to have significance scores <0, hence they were disqualified (Figure 3B). Through free combinations, there were 255 (2^8^-1) combinations for the remaining 8 DMGs. Following clustering using GMM and ROC analysis, combinations of Cluster 8 were found to have the optimal predictive performance (Figure 3C, Table S3). It was noted that the outlier combination MRPL51+SLK+PRC1 of Cluster 2 had the highest AUC, while the three genes were present in each model of Cluster 8. To obtain the simplified model, the combination MRPL51+SLK+PRC1 was eventually selected as the prognostic model. As a result, multivariate Cox regression was utilized to create a risk score model; further information can be found in Table S4. The C-indices of the model are 0.6341, 0.6771, and 0.6396, respectively, for the TCGA-LUAD, GSE42127, and GSE72094, indicating high prediction accuracy (Figure 3D). The three cohorts' tROC curves showed that the risk score model was highly successful in forecasting patients' OS (Figure 3E). A risk score was assigned to each patient in the TCGA-LUAD cohort, and based on the median score, high- and low-risk groups were identified. Patients in the high-risk group had a shorter OS time than those in the low-risk group, according to KM curves (p<0.0001) (Figure 3F). The riskplot displayed poor survival statuses in high-risk patients, and the expression of the three model genes was upregulated in the high-risk group (Figure 3G). The TCGA-LUAD cohort's 1-, 3-, and 5-year survival ROC-AUC values were 0.68, 0.64, and 0.58, respectively, indicating considerable predictive validity for the LUAD patient model (Figure 3H). Comparatively, the 1-, 3-, and 5-year survival ROC-AUC values for GSE42127 were 0.76, 0.71, and 0.69 (Figure S1A), while they were 0.64, 0.66, and 0.69 for GSE72094 (Figure S1B).

### The risk score model is independently prognostic for the survival of patients with LUAD

**Figure 4:**
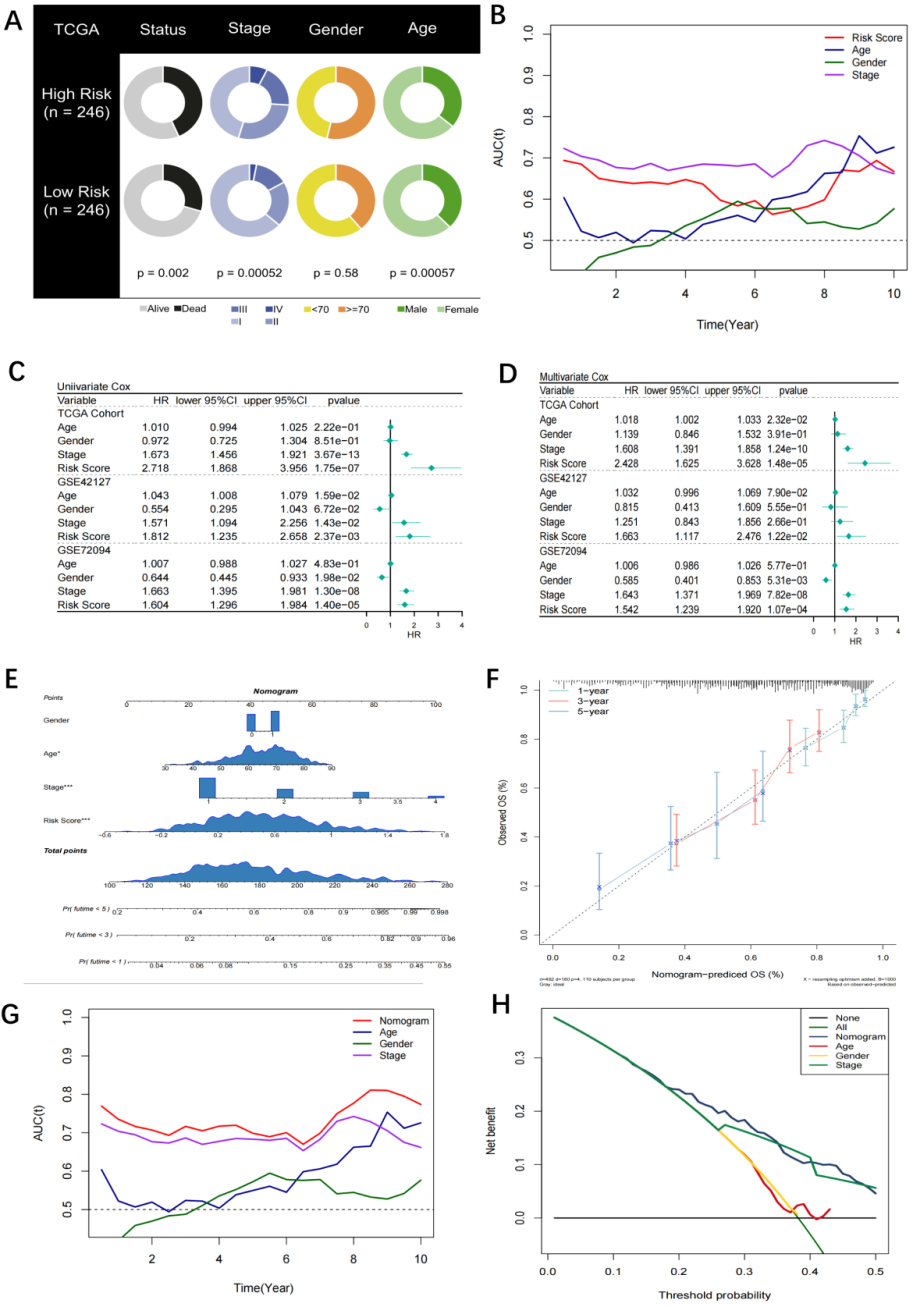
** Assessment of the riskscore model as an independent prognostic tool. (A)** Pie chart showing the difference of each variables in high- and low-risk groups identified by chi-square test. Except for gender, the other four variables were statistically difference between the two groups; **(B)** tROC curves for the riskscore and clinical characteristics in TCGA cohort. The riskscore was superior to gender, age and stage in prognosis with the best accuracy; **(C)** Univariate COX regression analysis for OS in TCGA and GEO cohorts; **(D)** Multivariate COX regression analysis for OS in TCGA and GEO cohorts; **(E)** Nomogram based on the riskscore and clinical characteristics; **(F)** Calibration curve for the nomogram; **(G)** tROC curves of the nomogram and clinical characteristics in TCGA cohort. The nomogram was superior to the clinical characteristics in prognosis with the best accuracy; **(H)** DCA curve for the nomogram. The nomogram mostly had satisfied performance in prognosis for 3-year survival under different thresholds.

A pie chart displaying the variations in risk score, age, stage, and other characteristics from the TCGA-LUAD, GSE42127, and GSE72094 studies reveals that significant disparities between the high- and low-risk groups can be seen in all parameters except sex (Figure 4A). The risk score had the greatest predictive accuracy compared to sex, age, and stage in Figure 4B. Additional univariate and multivariate Cox regression analysis showed that the risk score was a standalone prognostic factor for LUAD survival (Figure 4C, D). Similar results were also obtained in the subgroup analysis in the three cohorts (Figure S2). Accordingly, a nomogram was established (Figure 4E) and identified as accurate in prognosis through calibration analysis (Figure 4F). After evaluating the proportional hazard assumption, we confirmed that the nomogram model satisfies it. The variance inflation factor (VIF) was also computed, and we found that each variable's VIF was extremely close to 1, indicating that the nomogram's apparent multicollinearity may not truly exist. Additionally, the nomogram was superior to other variables as analysed by tROC curves (Figure 4G). Furthermore, the DCA curve showed that the nomogram was practicable for 3-year survival under most thresholds (Figure 4H).

### Functional enrichment of the three model genes

**Figure 5:**
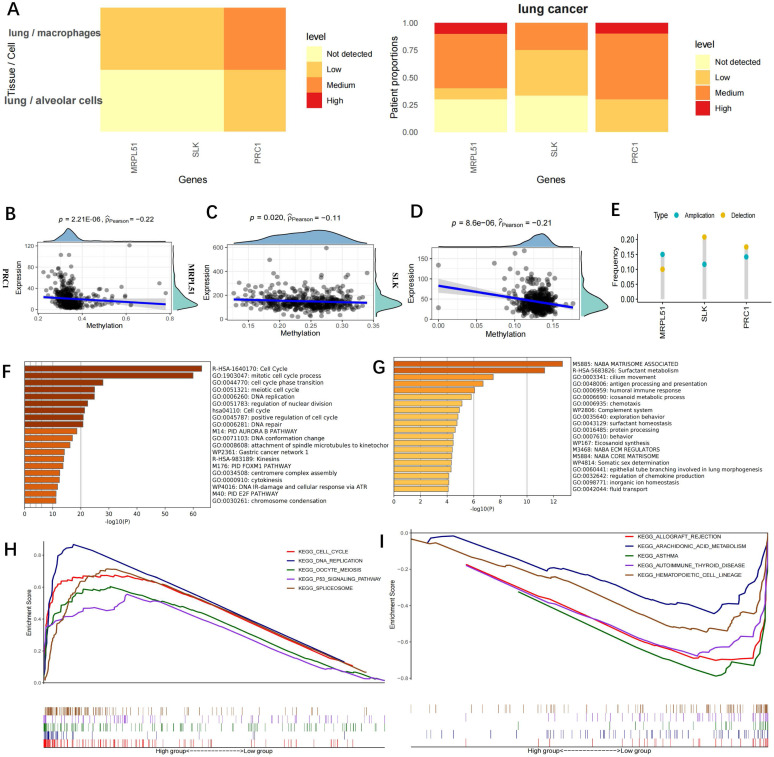
** Functional enrichment for the Risckscore. (A)** Immunohistochemistry data of the three model gene proteins in the HPA database. The three proteins exhibited up-regulated expression in lung cancer;** (B-D)** Correlation between the expression and methylation levels of PRC1 (B), MRPL51 (C), SLK (D); (E) CNV of the three model genes. Deletion was much more frequent in SLK and PRC1 genes while amplification was more frequent in MRPL51 gene; **(F-G)** Metascape functional enrichment analysis. The genes up-regulated in the high-risk group were mainly involved in cell cycle and DNA replication (Left), while the genes up-regulated in the low-risk group were mainly associated with antigen-presenting and immune response (Right); **(H-I)** GSEA enrichment analysis. High Riskcore was mainly involved in cell cycle and p53 signaling pathway (H), while low riskscore was majorly enriched in immune function-related pathways, such as asthma, autoimmune, hematopoiesis, autoimmune thyroid diseases (I).

Immunohistochemistry data of the three model gene proteins (MRPL51, SLK, PRC1) were retrieved from the HPA database and their expression was found to be upregulated in lung cancer (Figure 5A). The three model genes' expression and methylation levels had a negative correlation, according to the research (Figure 5B-D). DNA hypomethylation is among the earliest recognized epigenetic abnormalities in human tumours. At the whole-genome level, the majority of the genomes in somatic cells are highly methylated. In cancer cells, overall DNA hypomethylation is present, leading to chromatin rearrangement and decondensation. Additionally, metastatic tumours are more prone to be affected by DNA hypomethylation than primary tumours 21. Here, CNVs of the three model genes were analysed (Figure 5E). While MRPL51 tended to show CNV amplification, SLK and PRC1 were more likely to show CNV depletion. According to a metascape enrichment analysis (Figure 5F), the genes elevated in the low-risk group were mostly associated to antigen presentation and the immune response, while the genes raised in the high-risk group were generally linked to the cell cycle and DNA replication (Figure 5G). In GSEA enrichment analysis, the cell cycle and p53 signalling pathways were linked to high-risk scores (Figure 5H), whereas immune function pathways linked to low-risk scores included asthma, autoimmunity, haematopoietic cell lines and autoimmune thyroid disease (Figure 5I).

### Immune landscape in the high- and low-risk groups

**Figure 6:**
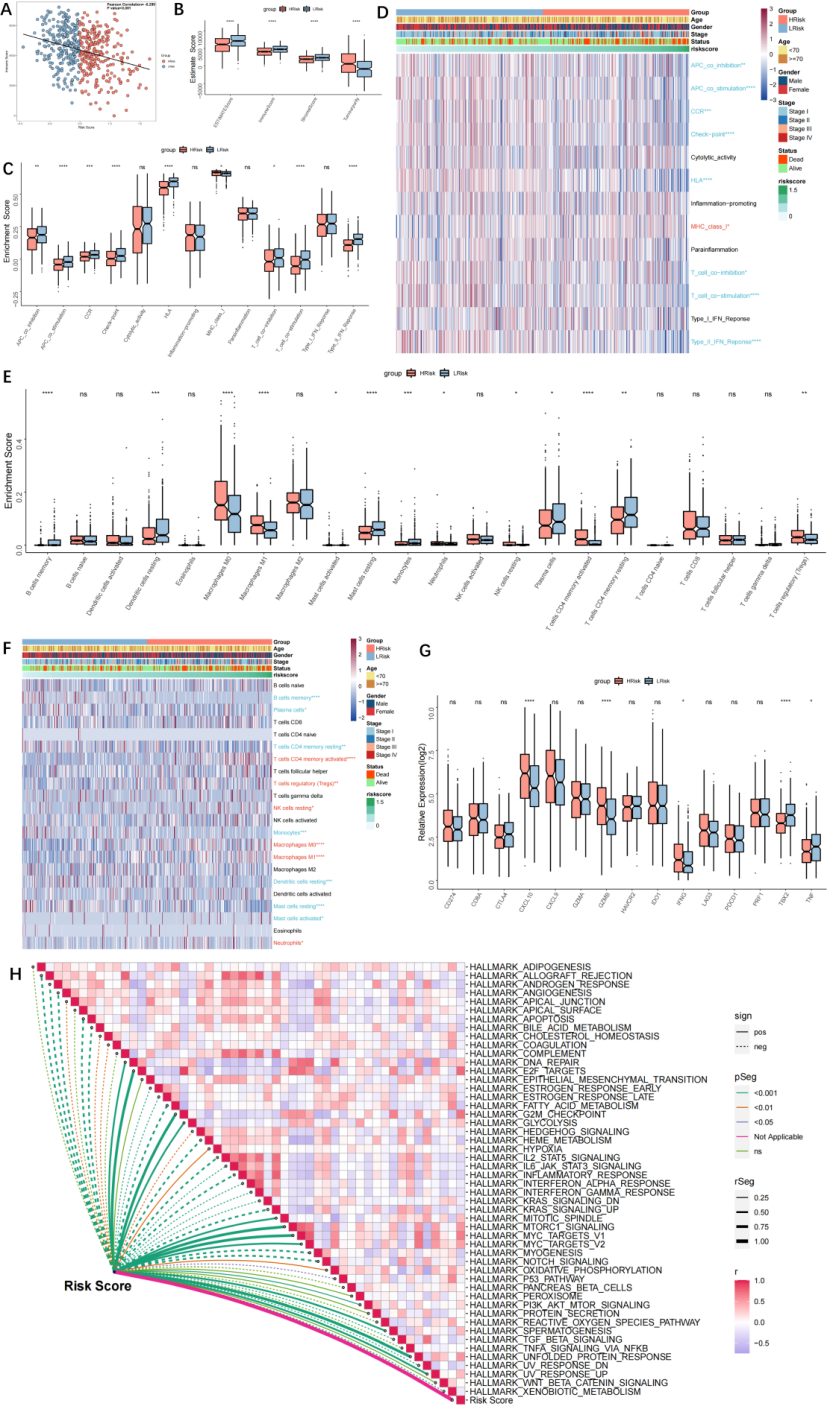
** Immune landscape in high and low-risk groups. (A)** Scatter plot showing a negative correlation between the riskscore and immune score (r=-0.299, p<0.001); **(B)** Box-plots showing the ESTIMATE scores in the high- and low-risk groups. The high-risk group had higher tumor purity, and the low-risk group had higher immune activity; **(C)** ssGSEA showing higher immune activity (including antigen-presenting and tumor immunity, except MHC1) in the low-risk group; **(D)** Immune landscape in high- and low-risk groups; **(E)** Box plot showing the difference in infiltration of immune cells in high- and low-risk groups; **(F)** Heatmap showing the infiltration of immune cells in high- and low-risk groups; **(G)** Box plot showing the difference in immune checkpoints scores in high- and low-risk groups; **(H)** Heatmap showing the correlation between the riskscore and Hallmark pathway activity; “Red name with * represents upregulated in high-risk score group, and blue name with * represents upregulated in low-risk score group; * P<0.05, **P<0.01, ***P<0.001”.

Risk score and immunological score had a negative correlation (r=-0.299, p<0.001, Figure 6A). Additionally, the high-risk group had greater tumour purity, while the low-risk group exhibited enhanced immune activity (Figure 6B). Immune pathways, with the exception of MHC1, were more active in the low-risk group, according to ssGSEA analyses. Furthermore, there was a statistically significant difference between the two groups in antigen presentation and tumour immunity (Figure 6C). A heatmap of the immunological landscape is shown in Figure 6D. The high- and low-risk groups showed distinct differences in the distribution of immune cells that invade tumours. Particularly, compared to the TME of the low-risk group, the TME of the high-risk group included considerably more Tregs, M0 macrophages, M1 macrophages, and activated CD4 T cells (Figure 6E). The function of CD4+ and CD8+ effector T cells, NK cells, and antigen-presenting cells may all be inhibited by Tregs in a number of ways, according to the available research, which results in an ineffective immune response and a poor prognosis. The low-risk group had higher levels of infiltrating B cells, DCs, mast cells, plasma cells, and latent CD4 cells. Antigen-presenting cells called DCs have drawn more interest recently. They aid in the recognition, preparation, and presentation of antigens as well as the start of the T cell-mediated immune response. The outcomes showed that patients in the low-risk group had more potent anti-tumour immunity. The topography of immunological cells in different groups is shown in a heatmap (Figure 6F). As immune checkpoint activity markers, CD274, CTLA4, HAVCR2, IDO1, LAG3, and PDCD1 were selected 22, whereas immune activity markers, CD8A, CXCL10, CXCL9, GZMA, GZMB, IFNG, PRF1, TBX2, and TNF were selected 12. The high-risk group had elevated levels of CXCL10, GZMB, and IFNG, whereas the low-risk group had downregulated levels of TBX2 and TNF (Figure 6G). The relationship between the risk score and the activation of the hallmark pathway was also analysed. Multiple pro-oncogenic pathways, such as E2F, MYC, and protein folding, as well as cell cycle processes, such as G2M and DNA REPAIR, were linked to the risk score, but not numerous metabolic and immune-related pathways, such as inflammatory response (Figure 6H). The findings also point to immunosuppression in the high-risk group and a more active immune system in the low-risk group.

### Genomic variations in high- and low-risk groups

**Figure 7:**
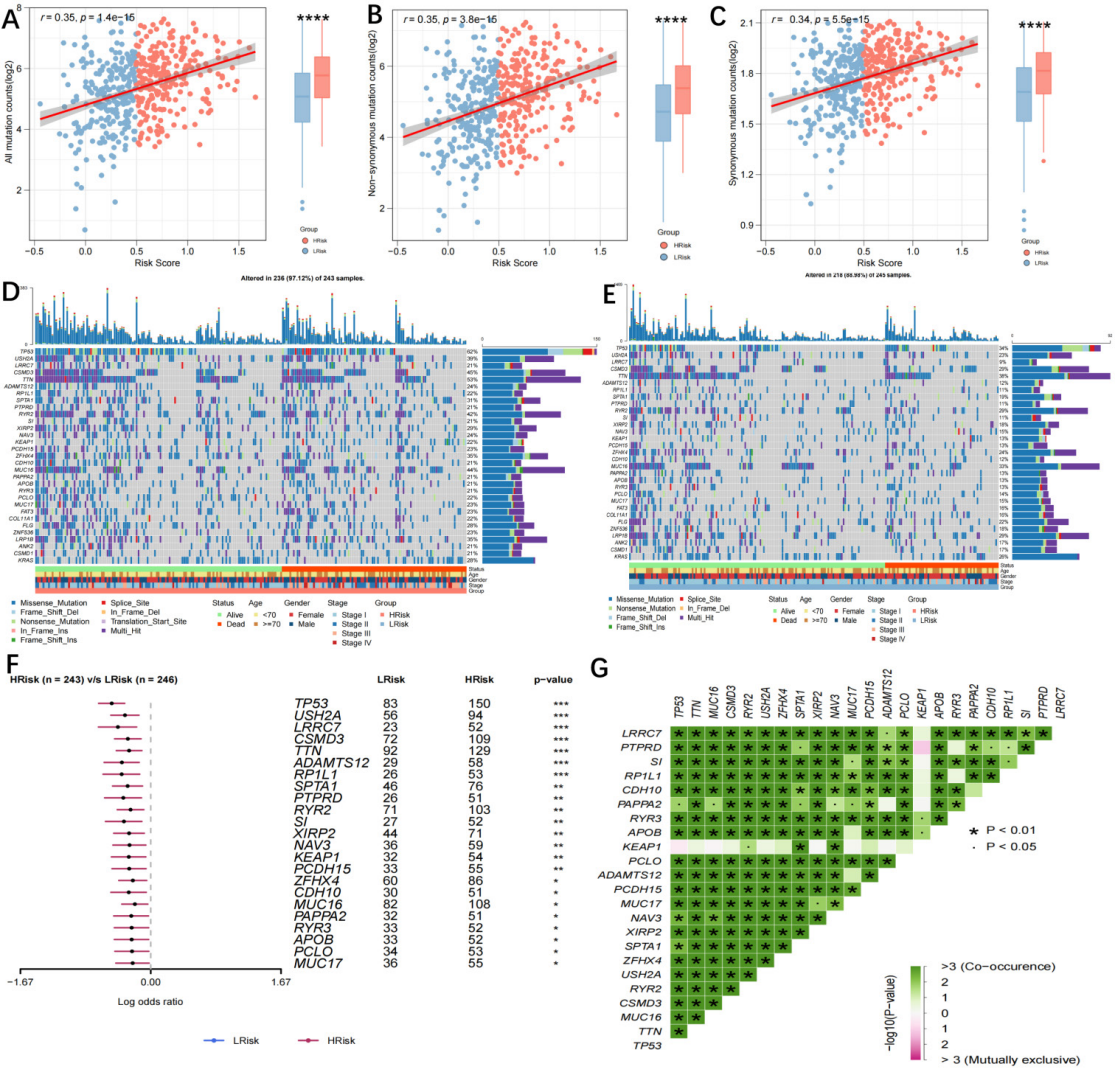
** Genomic variation in high- and low-risk groups. (A)** Scatter and Boxplot for all mutation counts (r=0.35, p=1.4e-15); **(B)** Scatter and Boxplot for non-synonymous mutation counts (r=0.35, p=3.8e-15); **(C)** Scatter and Boxplot for synonymous mutation counts (r=0.34, p=5.5e-15); **(D-E)** Waterfall plots for the mutations in 31 genes in high- (Left) and low-risk (Right) groups; **(F)** Forest map showing the 23 gene mutations of the highest frequency in high- and low-risk groups. Higher mutation frequencies were demonstrated in the high-risk group (*p < 0.05; **p < 0.01; ***p < 0.001); **(G)** Heatmap showing the colinearity of mutations in the 23 genes in high- and low-risk groups.

**Figure 8:**
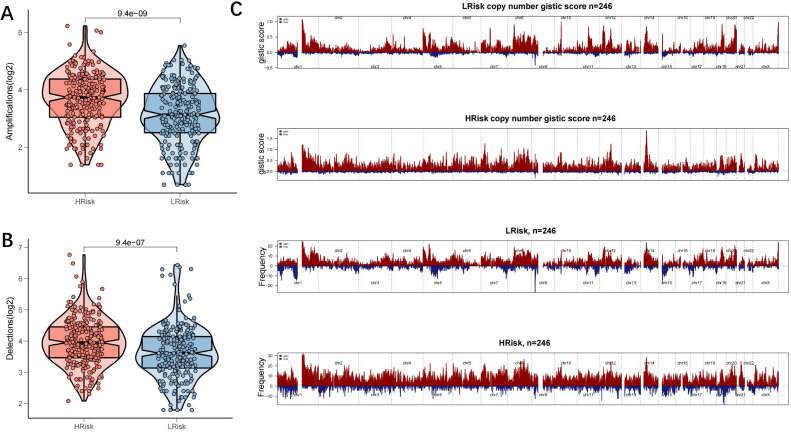
** Genomic mutation profile in high- and low-risk groups. (A-B)** Violin plots showing the amplification/deletion mutations in high- and low-risk groups; **(C)** CNV overview in high- and low-risk groups, including the logistic score and mutation frequency corresponding to different CNVs.

TMB is a novel biomarker that has received extensive attention in recent years. It is an indicator of the number of mutations in the tumour and its significance is in predicting the response of various tumours to immunotherapy by defining thresholds. In light of TMB's clinical importance, the relationship between TMB and the risk score was examined, and a significantly positive association was demonstrated (all mutation counts, r=0.35, p=1.4e-15; non-synonymous mutation counts, r=0.35, p=3.8e-15; synonymous mutation counts, r=0.34, p=5.5e-15) (Figure 7A-C). This indicated that high TMB does not definitely predict high immune activity. The 31 genes with TMB >30 mb were selected and intuitively displayed by Waterfall plots (Figure 7D-E). Table S5 contains a list of specific mutation data. A forest plot is also used to display the 23 mutated genes with the highest mutation frequency (p<0.05). The high-risk group showed increased mutation frequencies in all 23 genes (Figure 7F). The 23 genes were found to exhibit mutational collinearity (Figure 7G). Given that CNVs might lead to chromosomal alterations, the association between the risk score and CNV was further investigated. A noticeable increase in amplification and depletion was seen in the high-risk group (Figure 8A, B). The topography of CNV in the high- and low-risk groups is depicted in Figure 8C.

### Prediction of potential effective small-molecule drugs and chemotherapy resistance

**Figure 9:**
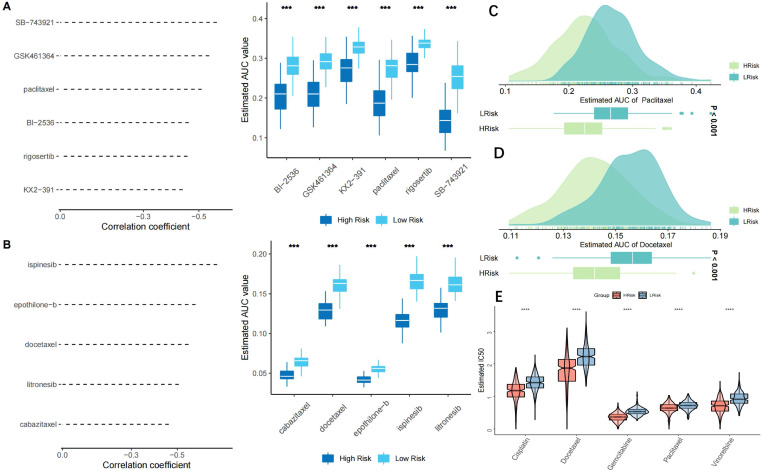
** Chemotherapy resistance analysis. (A-B)** A predictive model for drug resistance based on the CTRP (A) and PRISM (B) datasets. A total of 6 CTRP derivatives and 5 PRISM derivatives were obtained according to Spearman correlation between the riskscore and AUC score (Spearman's r < -0.35); **(C-D)** Drug sensitivity of two clinically common drugs, Paclitaxel (C) and Doxcetaxel (D); **(E)** Drug sensitivity of five common chemotherapeutics for lung cancer, Cisplatin; Docetaxel; Gemcitabine; Paclitaxel; Vinorelbine. IC50 values of the 5 agents were significantly higher in the low-risk group, indicating relatively poor sensitivity to chemotherapy in the low-risk group; *p < 0.05; **p < 0.01; ***p < 0.001.

Six CTRP2.0 derivatives (BI-2536, GSK461364, KX2-391, paclitaxel, rogosertib, SB-743921) and 5 PRISM derivatives (cabazitaxel, docetaxel, epothiloneb, ispinesib, litronesib) were retrieved from the CTRP2.0 and PRISM databases. In the low-risk group, all 11 derivatives displayed a higher AUC value (Figure 9A, B). Paclitaxel and docetaxel, two common clinic medications, were proven to be effective in patients with a high-risk (Figure 9C, D). Both paclitaxel and docetaxel are members of the taxane class of medicines that exhibit anti-tumor properties by preventing microtubule polymerization and reducing cancer cell mitosis and proliferation. Cell cycle and DNA replication were the most prominently affected genes in the high-risk group, in agreement with the results of the drug sensitivity test. The TCGA-LUAD cohort's sensitivity to five commonly used chemotherapeutic drugs (cisplatin, docetaxel, gemcitabine, paclitaxel, and vinorelbine) was then examined. The results showed that patients in the low-risk group were less susceptible to chemotherapy because their IC50 values for the five medications were significantly higher in the low-risk group than in the high-risk group (Figure 9E). The two GEO test datasets (GSE42127 and GSE70294) likewise produced similar outcomes (Figure S4A, B). There were 73 DEGs deemed to represent possible small-molecule targets between the high- and low-risk groups, and 37 chemical pathways were identified (Figure S3A). The Genomics of Drug Sensitivity in Cancer (GDSC) database and the Cancer Therapeutics Response Portal (CTRP) were used to evaluate the sensitivity and resistance of SLK, MRPL51, and PRC1-targeting drugs. According to the results of our Pearson's correlation study, drug sensitivity to docetaxel, 17-AAG, and afatinib in the GDSC database was inversely linked with SLK expression based on the IC50 value (Figure S3B). The CTRP database revealed a negative association between the level of SLK expression and the IC50 value and drug sensitivity to sarcacatinib and erlotinib (Figure S3C). SLK was the model gene that drug sensitivity study indicated was the ideal target.

### Prediction of immunotherapy response

**Figure 10:**
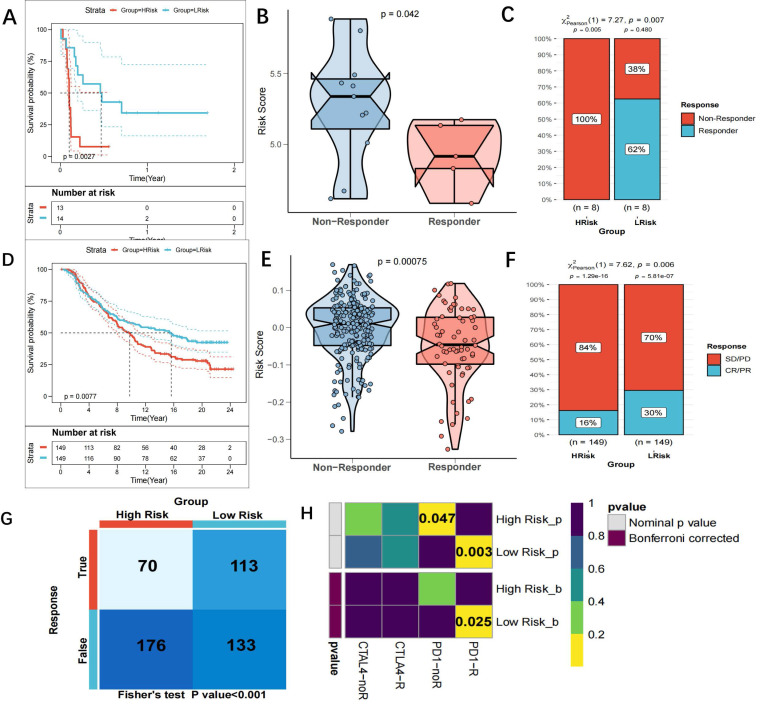
** Prediction of response to immunotherapy. (A)** KM curves showing the poorer OS in NSCLC patients receiving anti-PD1 therapy of the high-risk group in GSE135222 (p=0.0027); **(B)** Violin plots showing significantly decreased Risckscores in patients responding to immunotherapy in GSE126044 (p=0.042); **(C)** Graph plots showing higher response rates to anti-PD1 therapy in low-risk group that high-risk group (p=0.007 in chi-square test); **(D)** KM curves for the high-risk and low-risk groups in the IMvigor 210 cohort; **(E)** Violin plots showing the significant difference in the riskscore between patients responsive and irresponsive to immunotherapy in the IMvigor 210 cohort; **(F)** Clinical response rates to immunotherapy, including complete response [CR] /partial response [PR] and stable [SD] /progressive disease [PD], in high- and low-risk groups in the IMvigor 210 cohort; **(G-H)** scatter and boxplot for TMB(G) and neoantigens(H) between high and low-risk group. It demonstrated that risk score was inversely associated with TMB and neoantigens, and TMB and neoantigens increased significantly in the low-risk group. (*p < 0.05; **p < 0.01; ***p < 0.001); **(I)** TIDE scores corresponding to immunotherapy responses of high- and low-risk groups in the GEO cohort;. A higher sensitivity to anti-PD1 therapy was demonstrated in the low-risk group; **(J)** Subclass mapping for predicting sensitivity to anti-PD1 and anti-CTLA4 treatment in patients belonging to the high-risk and low-risk groups. Patients in the low-risk group were more sensitive to anti-PD1 therapy (chi-square test, FDR=0.025).

As previously mentioned, there might be a link between the risk score and immunotherapy response. The GSE135222 dataset of NSCLC patients undergoing anti-PD-1 therapy was examined to more thoroughly assess this, and we discovered that those with a high-risk score had shorter survival times (p=0.0027) (Figure 10A). A reduction in the risk score was observed in patients who responded to immunotherapy in the GSE126044 dataset (Figure 10B). Furthermore, the chi-square test demonstrated that the low-risk group reacted to anti-PD-1 treatment at a greater rate than the high-risk group (p=0.007) (Figure 10C). In the IMvigor210 dataset, patients with a high-risk score had a worse survival result (Figure 10D), and patients who had anti-PD-L1 treatment responses had lower scores (p=0.0075) (Figure 10E). The low-risk group had a greater response rate to anti-PD-L1 treatment, according to the chi-square test (Figure 10F). Each patient's neoantigens and TMB data are included in the IMvigor210 cohort. In an attempt to comprehend why patients with a low-risk score are more receptive to immunotherapy, we evaluated the relationship between risk score, neoantigens, and TMB. In Figure 10 G-H, we discovered that TMB and neoantigens had an inverse relationship with risk score and considerably increased in the low-risk group. TIDE scores were calculated and showed that patients in the low-risk group in the TCGA cohort were more sensitive to anti-PD-1 therapy (p=0.006) (Figure 10I). Subclass mapping (FDR=0.025) also supported this (Figure 10J). The GSE42127 and GSE70294 datasets yielded similar results (Figure S4C-F). Taken together, the data suggest that the risk score model is a strong tool that may help lung cancer patients make treatment decisions. Patients with a low-risk score may also benefit more from immunotherapy and have a better survival outcome.

## Discussion

We studied DNA methylation markers in CTCs for the first time and connected them to patient prognoses, immunological modulation, and treatment effectiveness in LUAD for the first time. According to our results, the DMGs-based risk model performed admirably in both the training and external validation datasets. The high-risk group had significantly lower survival rates than the low-risk group. This discovery revealed that DMGs might be useful in LUAD precision medicine. First, we found DMGs related with metastasis from DEGs retrieved from GSE74639 (CTC and primary tumor samples) and TCGA-LUAD (metastatic and non-metastatic samples). A predictive signature comprising MRPL51, SLK, and PRC1 was identified using univariate Cox regression, log-rank test, and band random forest analysis. The multivariate Cox regression method was then used to create the risk score model. In the TCGA cohort, the ROC-AUC values for 1-, 3-, and 5-year survival were 0.68, 0.64, and 0.58, respectively. This result showed that the model may predict the survival of LUAD patients to some extent. The model also performed well in the datasets GSE42127 (1-year, 0.76; 3-year, 0.71; 5-year, 0.69) and GSE72094 (1-year, 0.64; 3-year, 0.66; 5-year, 0.69).

Patients in the TCGA-LUAD cohort were divided into two groups based on the median risk score in order to investigate the biological processes associated with it. The high-risk group's increased genes were mostly involved in the cell cycle and DNA replication. A recent study has found that abnormal cell cycle regulation is strongly linked to cancer and development 23. Increased cell cycle activity harms anti-tumor immunity. Inducing cell cycle arrest might be a potential method of inhibiting cancer cell proliferation and a promising therapeutic strategy to tumor growth 24. Interestingly, it was shown that the genes increased in the low-risk group play a crucial role in antigen presentation and immune response. A lower risk score was shown to be associated with greater anti-tumor immunity and cell-killing abilities. Furthermore, the GSEA results showed that a high-risk score was connected with cell cycle and p53 signaling pathways, while a low-risk score was associated with immune-related pathways such as asthma, autoimmunity, haematopoietic cell lines, and autoimmune thyroid disease. This meant that tumors in high-risk patients were actively growing, whereas individuals with low-risk scores exhibited immunological hyperfunction. This indicated that tumors in patients with a high-risk score were actively proliferating, while those with a low-risk score had immunological hyperfunction. Immunotherapy resistance is more frequent in patients with a high-risk score, which may lead to poor survival results.

The paradigm of cancer treatment was completely altered by the development of immunotherapy. This improved the survival time of many LUAD patients and gave hope to people who were previously incurable 25. However, some patients are still immune to immune checkpoint blockers and cannot benefit from them (ICBs). Some ICB-resistant patients do not react to immunotherapy (innate resistance). Others initially react to ICB but develop acquired resistance (insensitivity) as the illness worsens 26. Immune evasion, which tumor cells use to avoid immune monitoring and elimination, is one of the primary mechanisms causing immunotherapy resistance 27,28. The main factor affecting TME is the level of immune cell infiltration. In this abnormal circumstance, Cancer antigens may cause immune responses to be suppressed in the TME. Extracellular chemicals, immune cells, stromal cells, cytokines, and chemokines comprise the TME, which is a dynamic and complex system 29.

The TME has the potential to have both favorable and unfavorable effects on tumorigenesis 30. The TME may vary continuously as the tumor develops. Both groups' immunological landscapes were examined. Patients in the low-risk group were discovered to have higher immunological scores and stronger immune checkpoint activation, which suggested a more robust immune system. We also found that the high-risk group had significantly more Tregs, M0 macrophages, M1 macrophages, and activated CD4+ T cells in the TME than the low-risk group did. Numerous studies have shown that Tregs may limit the function of CD4+ and CD8+ effector T cells, NK cells, and antigen-presenting cells in a number of ways, which eventually leads to an ineffective immune response and a poor prognosis. In addition, in the low-risk group, more infiltrating B cells, DCs, mast cells, plasma cells, and resting CD4 cells was observed. DCs are important antigen-presenting cells that can boost T cells' ability to fight tumors. The findings showed that low-risk patients mounted more powerful anti-tumor immunity. The TME can be categorised into two categories: hot and cold, depending on the level of T cell infiltration and the expression of particular cytokines. In line with this, the tumors in the high-risk group in this study were classified as “cold” with immunosuppression, which may be related to a bad prognosis. The tumors in the low-risk group, however, were deemed “hot” and had immune hyperfunction linked to a positive prognosis. Our conclusion is supported by earlier studies, which found that the immune-cold subtype or the fatigued subclass were predictors of poor survival 31. Additionally, according to certain research, immune-related genes were substantially expressed in hot tumours, which is consistent with the high immune score seen in these tumors, and the high immune score group had a better prognosis 32. Distinguishing between hot and cold tumours and transforming cold tumours into hot tumours will improve the anti-tumour effect of immunotherapy and bring a breakthrough to immunotherapy.

It has been reported that TMB (tumor mutational burden) is a biomarker of immunotherapy response 33, with higher TMB predicting better immunotherapy response 34. Our results demonstrated a higher TMB in patients with high risk. However, as previously noted, individuals in the high-risk group had lesser immunological activity, indicating that a high TMB did not always indicate a high immunogenicity. Further investigation found that mutations in the 23 genes were the primary cause of the increased TMB in the high-risk group. In the high-risk group, these genes exhibited a high frequency of comutations and elevated TMB. Further research is needed to determine if these comutations affect patients' responses to immunotherapy.

Given the importance of CNV in chromosome variation, we looked into the relationship between the risk score and CNV further. Patients with a high-risk score experienced more amplification and depletion events than patients with a low-risk score. Somatic rearrangements are oncogenic, can increase tumor heterogeneity, and correlate with immunotherapy resistance, according to research. These findings suggest that patients at high risk may not respond well to immunotherapy, whereas patients at low risk may be more sensitive. According to McGranahan et al. 35, a heterozygous deletion in the HLA gene may be a major cause of ineffective immunotherapy in NSCLC patients with a high TMB. They also stated that the efficacy of TMB-guided immunotherapy is limited.

Previous studies strongly suggested higher sensitivity to immunotherapy in patients at a low risk 36. In line with these findings, the TIDE score and subclass mapping method used among the present investigation demonstrated increased sensitivity to anti-PD-1 medication in patients in the low-risk group. Three more independent cohorts were analyzed, and the results showed that patients in the low-risk category were more responsive to anti-PD-L1 medication and had longer survival times. This provides more convincing evidence. When the risk score was high, NSCLC patients receiving anti-PD-1 medication in the GSE135222 cohort had a worse survival outcome. Patients with NSCLC who responded to anti-tumor immunotherapy belonged to the GSE126044 group and had a lower risk score. Additionally, in the IMvigor210 cohort, patients with metastatic urothelial carcinoma who were engaged in a large-scale phase II study showed a greater response rate to anti-PD-L1 immunotherapy and a longer survival time. Our predictive model performed well in different cancer types despite being built using LUAD data. This further demonstrated our model's stability and dependability and underlined its broad application in a variety of cancer types.

Neoantigens and TMB information for each patient are also included in the IMvigor210 cohort. As a consequence, we analyzed the relationship between TMB, neoantigens, and risk score. We discovered that TMB and neoantigens had an inverse relationship with risk score and that they considerably increased in the low-risk group. And in order to trigger anti-tumor immunity, immunotherapy mostly relies on CD8+ T cells that can detect mutant antigens specific to tumors. Additionally, additional somatic mutations will increase the production of possible new antigens. Therefore, additional TMB and neoantigens may boost the immunotherapy sensitivity of patients with low risk scores. Anti-tumor immunotherapy may generally have a greater survival benefit for patients in the low-risk group. Our model can accurately identify patients at high risk for immunotherapy resistance and thus guide immunotherapy. Finally, using the CTRP2.0 and PRISM datasets, the current study examined chemotherapy sensitivity in patients in the two groups. The results showed that patients in the high-risk group were more sensitive to paclitaxel and docetaxel, which was in line with the Metascape enrichment analysis, which showed that the high-risk group's elevated genes were mostly involved in DNA replication and the cell cycle. The findings of the study demonstrate the value of the risk score as a tool for LUAD patient treatment management.

Nevertheless, this study still has some limitations that need to be resolved. First, the methylation data were derived from TCGA rather than the data of the CTC samples from GEO. Second, although we assessed and validated the prognostic performance of the model by evaluating sensitivity to chemotherapy and immunotherapy using multiple algorithms, more prospective studies and clinical data are required for further validation. Third, further *in vivo* and *ex vivo* research is needed to explore the biological roles of the differentially methylated genes in LUAD.

In conclusion, this research created a predictive risk score model for LUAD based on the DEGs of CTCs and identified variations in biological function, TME, genetic variation, and clinical outcomes between individuals at high and low risk. We also learned that the sensitivity to chemotherapy and immunotherapy may be predicted using the risk score model. The results of the present investigation may improve LUAD clinical care and extend knowledge of precision medicine.

## Supplementary Material

Supplementary figures.

Supplementary tables.

## Figures and Tables

**Table 1:** Baseline clinical characteristics of three major cohort

Characteristics	TCGA Cohort	GSE42127	GSE72094
n	492	132	398
Follow up time (mean (SD))	2.48 (2.44)	4.12 (2.64)	2.17 (1.10)
Status (%)			
Alive	312 (63.4)	90 (68.2)	285 (71.6)
Dead	180 (36.6)	42 (31.8)	113 (28.4)
Gender (%)			
Female	267 (54.3)	65 (49.2)	222 (55.8)
Male	225 (45.7)	67 (50.8)	176 (44.2)
Stage (%)			
Stage I	268 (54.5)	89 (67.4)	255 (64.1)
Stage II	119 (24.2)	22 (16.7)	69 (17.3)
Stage III	80 (16.3)	20 (15.2)	58 (14.6)
Stage IV	25 ( 5.1)	1 ( 0.8)	16 ( 4.0)
Age (%)			
<70	307 (62.4)	80 (60.6)	184 (46.2)
≥70	185 (37.6)	52 (39.4)	214 (53.8)
