# Supplementary material for: Development and Validation of a DNA Methylation-related Classifier of Circulating Tumour Cells to Predict Prognosis and to provide a therapeutic strategy in Lung Adenocarcinoma
Source: Int J Biol Sci. 2022 Aug 1;18(13):4984–5000. doi: 10.7150/ijbs.75284 (PMC9379404; doi:10.7150/ijbs.75284)
Supplement: Supplementary file 1 — Supplementary figures. [file ijbsv18p4984s1.pdf]

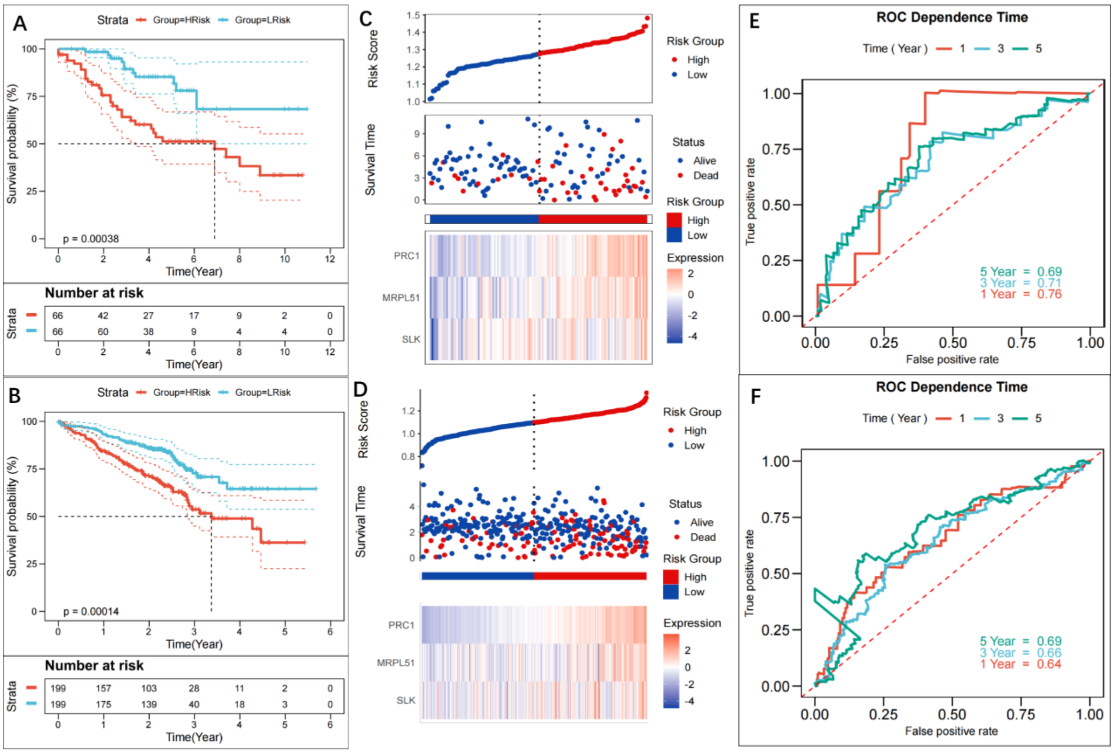

Figure S1. Survival analysis in GSE42127 and GSE72094 datasets.

(A-B) KM survival curves for the high- and low-risk groups in the GEO cohorts; (C-D) Survival status of patients and expression of marker genes in GEO cohorts; (E-F) 1-, 3-, and 5-year ROC curves for the riskscore in GEO cohorts.

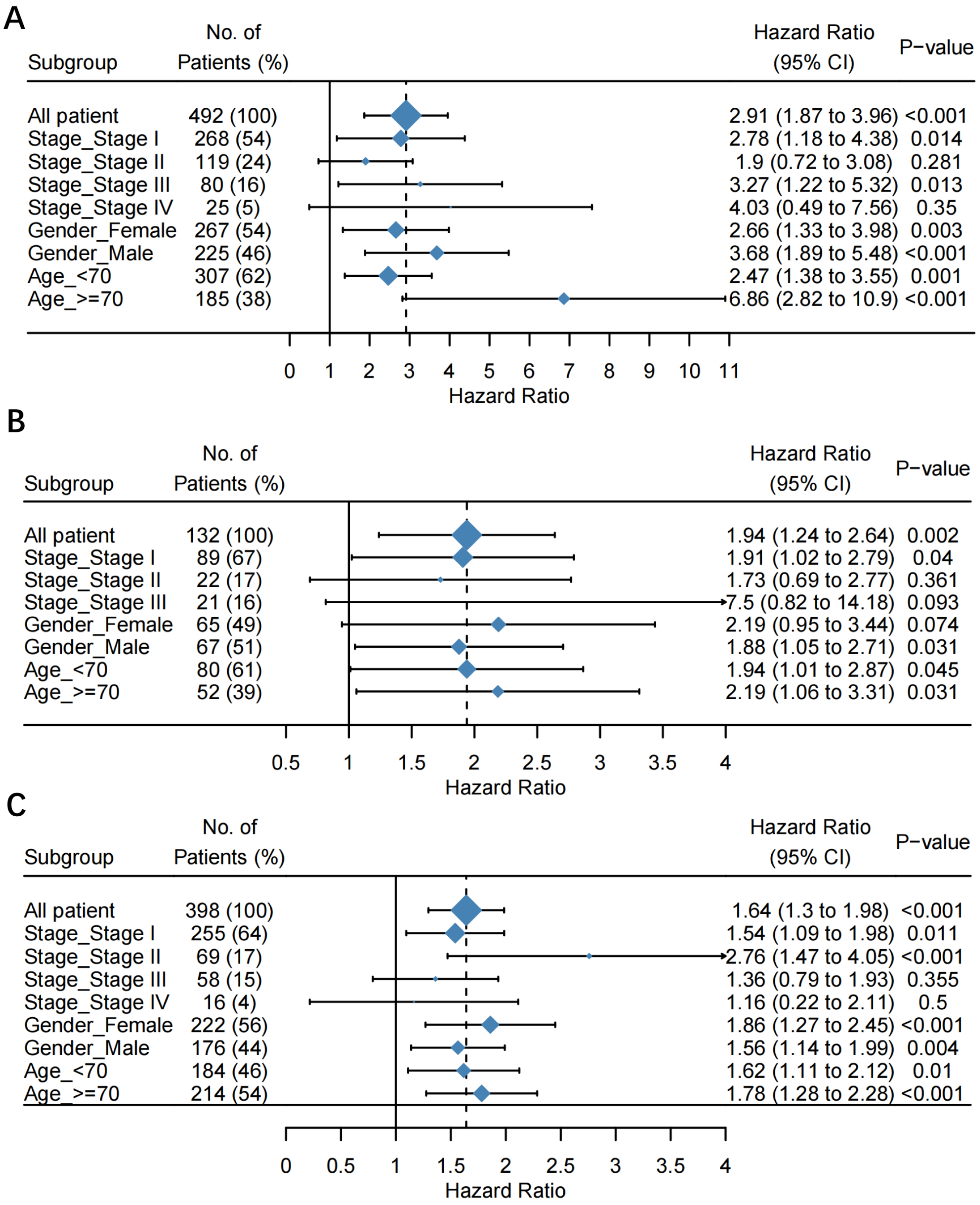

Figure S2. Forest map for the prognostic performance of the riskscore in TCGA (A), GSE42127 (B), and GSE72094 (C) cohorts.

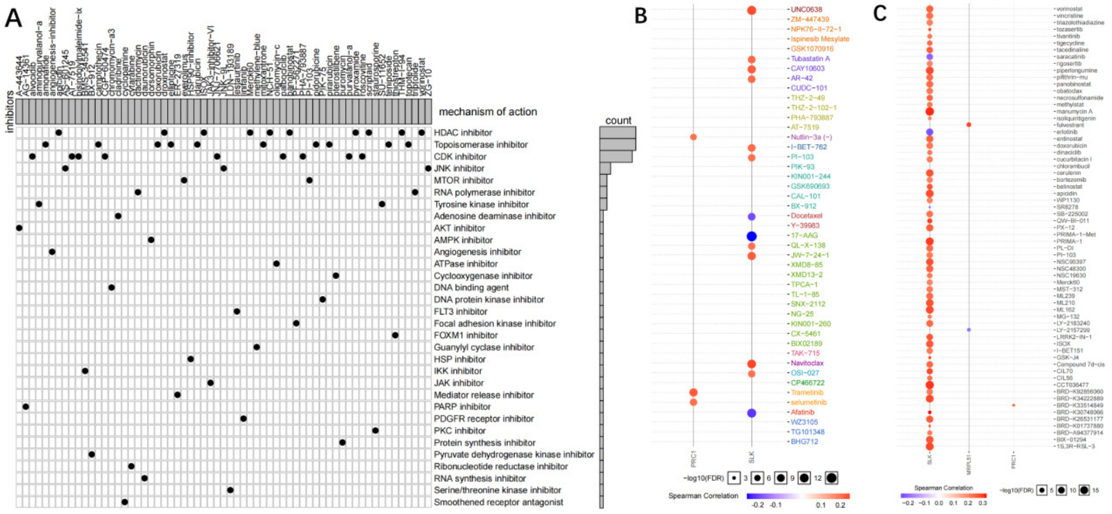

Figure S3. Analysis of potential small-molecular drugs and sensitivity of targeted drugs. (A) 73 small-molecular drugs-pathway network using MoA analysis; (B) Drug sensitivity analysis of gene sets based on Genomics of Drug Sensitivity in Cancer (GDSC) IC50 drug data; (C) Drug sensitivity study of a gene set based on Cancer Therapeutics Response Portal (CTRP) IC50 drug data. Pearson's correlation shows the relationship between gene expression and drug sensitivity. Negative correlations were represented by blue bubbles, whereas positive correlations were represented by red bubbles; the darker the hue, the stronger the link. The size of the bubble was connected to the FDR importance. The dark outline denotes a 0.05 FDR. Only the top 30 medicines were considered.

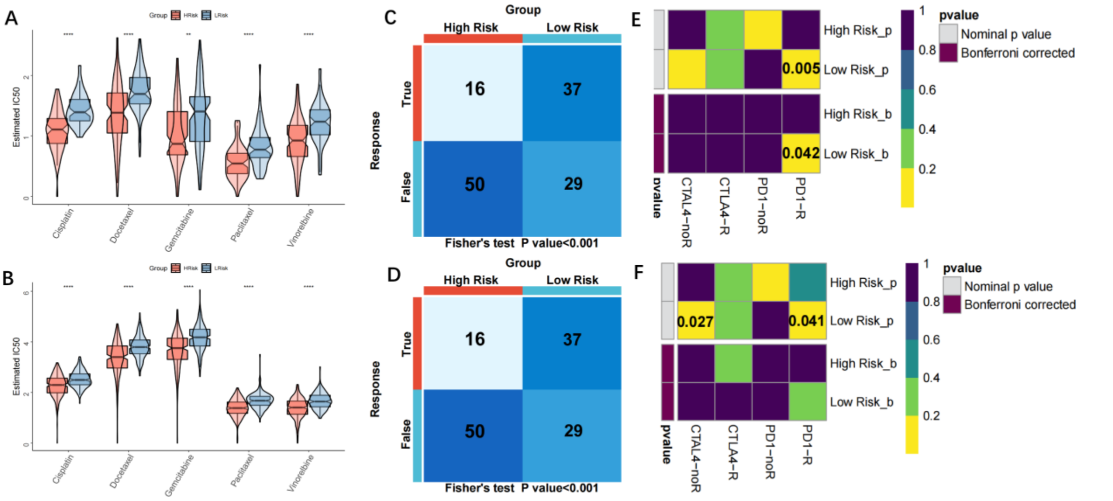

Figure S4. Validation of the treatment decisions in GSE42127 and GSE70294 cohorts. (A-B) Sensitivity of five common chemotherapeutics in high- and low-risk groups; (C-D) Immune response in high- and low-risk groups using subclass mapping; (E-F) Immune response in high- and low-risk groups based on TIDE scores; *p < 0.05; **p < 0.01; ***p < 0.001.
